# Impact of a biomedical research project on the human capital development of emerging researchers

**DOI:** 10.17159/sajs.2018/20170090

**Published:** 2018-03-27

**Authors:** Christabelle S. Moyo, Joseph Francis, Pascal O. Bessong

**Affiliations:** 1 Department of Microbiology, University of Venda, Thohoyandou, South Africa; 2 Institute for Rural Development, University of Venda, Thohoyandou, South Africa

**Keywords:** skills, knowledge, community-based research, rural-based university

## Abstract

Various evaluative studies have been carried out to obtain the views of multiple stakeholders involved in community-based biomedical research projects. However, rarely have the viewpoints of postgraduate students and junior faculty involved in such initiatives been explored. Thus, the aim of this study was to examine the views of postgraduate students and junior faculty at a rural-based university on the effect of a longitudinal biomedical research project on their acquisition of relevant skills. In-depth interviews and a focus group discussion were conducted. The thematic content analysis technique was used to analyse the qualitative data. Both postgraduate student and junior faculty groups indicated that they had acquired considerable research skills and knowledge; gained experience; were exposed to practical reality; and strengthened their interpersonal skills and general personal development. However, some respondents highlighted that they still believed that training in data analysis and exposure to new laboratory techniques would have strengthened their individual capabilities to conduct cutting-edge research. The results of this study highlight the need for community-based biomedical researchers to equip members of their teams with the skills and knowledge that will help them achieve their academic and career goals.

## Introduction

Various evaluative studies have been conducted in different parts of the world, which aim to elicit multiple stakeholder viewpoints and experiences. Among these have been studies focused on community-based biomedical projects or programmes.^1-4^ Results obtained in some of the aforementioned studies have highlighted the importance of effective engagement of study participants in projects carried out in their localities. Engagement can be partially achieved through creating communication platforms in which project participants share their experiences and thoughts regarding research projects conducted in their communities. Through such interactive processes, researchers and development practitioners would gather information that might help in planning and implementing future initiatives. Although numerous studies on community-based biomedical research projects have been conducted, the viewpoints of postgraduate students and junior faculty involved in such initiatives have rarely been explored. Exploring the perspectives of postgraduate students and junior faculty might assist research project leaders to better plan similar initiatives and to take some corrective action when the need arises. Moreover, studies of this nature are likely to reveal the effects of community-based biomedical research projects beyond merely generating scientific knowledge. In addition, exploring the ‘hidden’ issues of biomedical research projects has the potential to bring about a holistic understanding of community-based projects and the benefits accruing to members of study teams. The preceding arguments necessitated carrying out the current study. In this study, the perspectives of postgraduate students and junior faculty on the effects of a biomedical community research project on their human capital development were explored.

Marimuthu et al.^5^ define human capital as ‘processes that relate to training, education and other professional initiatives in order to increase the levels of knowledge, skills, abilities, values and social assets of an employee which will lead to the employee’s satisfaction and performance and eventually on a firm’s performance’. The Organisation for Economic Co-operation and Development^6^ concurs with these views and defines human capital as knowledge, skills, competencies and attributes embodied in individuals that assists in the creation of personal, social and economic well-being. Included in this definition are non-economic attributes such as motivation, communication, inter-personal skills, perseverance, self-discipline, behaviour, and physical, emotional and mental health of individuals.^6^ Thus, investing in human capital is a strategy that helps increase economic returns.^5^ Human capital can be acquired either through formal early childhood learning, primary and high schooling, tertiary and post-tertiary training, adult education or through informal education such as family, peers or on the job learning.^6^ It is evident from these perspectives that education is a primer for development. In the current study, human capital refers to the acquisition of skills and knowledge, experience and exposure, interpersonal skills and personal development through informal post-tertiary training. Human capital attributes such as motivation, confidence building and working independently are also included in the definition.

Human capital theory, which is rooted in the works of Schultz and Becker^7^, anchored the current study. Becker^8^ argues that human capital is linked to economic growth and that education, training and health are the crucial elements of human capital. Becker^8^ also points out that education, training and health are important investments that precipitate human capital, because they assist in raising income, improving well-being and enhancing the good habits of an individual over their lifetime. Education and training increase the chances of being employed.^9^ When individuals acquire relevant skills and knowledge, they often make better informed decisions about their lives, such as those relating to career path, financial investments, how to live amicably with others, eradicating poverty, nutrition, healthy lifestyles and various other ways of improving their well-being.^5,6,8^ Nhamo and Nhamo^10^ postulate that education enhances the potential earning capacity of individuals, given that enlightened individuals tend to be more productive and are able to perform more complex tasks. Mincer^11^ lends weight to this view and states that employers set better rewards for employees with higher qualifications because they are more skilled and, in general, are more productive. It is also worth noting that globalisation is now heightening competition for skilled human resources.^12^ Personnel with higher educational qualifications, relevant expertise and experience can be recruited from any part of the world. Thus, investments in human capital are crucial.

The current study was embedded in the ‘Etiology, Risk Factors, and Interactions of Enteric Infections and Malnutrition and the Consequences for Child Health and Development’ (MAL-ED) South Africa project, which was initiated in 2009 and undertaken in the Dzimauli community of the Vhembe District in the Limpopo Province of South Africa. Bessong et al.^13^ and the MAL-ED Network Investigators implemented the MAL-ED project. Postgraduate students and junior faculty from a rural-based university were recruited and trained to collect data on feeding habits, childhood diseases, vaccinations and biospecimens, among others. As highlighted earlier, the aim of the current study was to interrogate the perspectives of postgraduate students and junior academic staff who participated in the MAL-ED project with respect to the impact of the project on their human capital development.

## Description of the study site

The study was conducted between 1 January and 28 February 2016 at a rural-based university in Thohoyandou in the Limpopo Province of South Africa. The study site lies approximately 180 km north of Polokwane, the capital city of the Limpopo Province.

## Research methodology

### Research design, population and sampling

A case study research design was adopted. This study was exploratory and qualitative. According to Kothari^14^, qualitative research entails obtaining subjective assessments of attitudes, opinions and behaviour. Santha et al.^15^ also explain that this type of research is concerned with the opinions, experiences and feelings of individuals, implying that subjective data are collected. It was deemed appropriate to adopt the qualitative research approach in the current study because it allowed the investigators to interrogate the perceptions of participants with a view of getting deeper insights on their acquisition of relevant skills.

Out of a target population of 34 people, 15 postgraduate students (pursuing honours, master’s and doctoral studies), 7 junior faculty and 3 heads of departments (HoDs) voluntarily participated in the study. Of the 25 people who volunteered to participate in the study, 23 of them had been involved in the initial phase (24 months of follow-up) of the MAL-ED project. The remaining two participants were HoDs who had not been directly involved in the project but supported junior faculty from their departments to actively participate. It was deemed necessary to obtain their views on the academic performance of the members of staff from their departments who had been involved in the MAL-ED project. Out of the 15 postgraduate students who participated in the study, 11 took part in the individual face-to-face interviews. The remainder were interviewed telephonically because work-related commitments made it impossible for them to present themselves for scheduled interview sessions.

Open invitation letters were sent to the postgraduate students and junior faculty requesting them to participate in the current study. The purpose and benefits of participating in the study were clarified in the invitation letters. Furthermore, the letters revealed that participation in the study was not compulsory, meaning that they were free to withdraw at any point. The participants were 25–51 years old. The highest academic qualification was a doctorate, with the lowest being a diploma.

The University Research and Ethics Committee approved the study protocol (protocol no: SMNS/16/MBY/01/0701). This paved the way for the study to be conducted. Before commencement of the interviews, postgraduate students, junior faculty and their HoDs were made aware of the ethics to which the study would adhere. The right to anonymity, confidentiality and the freedom to withhold sensitive information if they so wished were specifically highlighted. Furthermore, permission to use an audio recorder during both the face-to-face in-depth interviews and the focus group discussion was sought from the participants. Signed informed consent was obtained from all the participants prior to their participation in the study.

### Pilot testing research instruments

Interview guides were pilot tested with four respondents who were part of the target population. However, the latter were not included in the final study sample. Pilot testing was done in order to check the reliability of the data collection tools. This testing involved checking how long it took to conduct each interview, clarity of the questions and ease of understanding the interview questions.^16^ The interview guides were given to other colleagues not involved in the study but with relevant knowledge and expertise for their comments. After the pilot testing exercise, the research instruments were re-worked to ensure that the questions were clearer, more precise and could be administered within the stipulated time frame.

### Data collection and analysis

Data were collected through individual interviews and a focus group discussion. In-depth interview schedules guided the data collection. The interview schedule for postgraduate students and junior faculty sought to determine the relevant skills and knowledge that the participants had acquired as a result of their participation in the MAL-ED project. The principal researcher ensured that the individual interviews were held in a quiet environment and that they did not interfere with the academic commitments of the participants. On average, each interview was concluded within 50 minutes. The participants’ responses regarding the benefits of the project were categorised into four identified categories derived from the themes of the study. However, the responses were so varied that data saturation was not reached.

After the individual interviews were complete, a focus group discussion was held. The group discussion involved 10 of the 22 postgraduate students and junior faculty who had participated in the individual interviews. The remaining 12 persons failed to participate in the focus group discussion because of other commitments. The focus group was designed to establish if group dynamics would elicit different perspectives from those obtained in the individual interviews. The participants chose their own chairperson and scribe from within the group. This approach enabled the group to select an impartial person who would allow them to express their views freely. The chairperson facilitated the group discussion and the scribe recorded the responses once there was consensus on an issue. The principal researcher ensured that there was order in the engagements. The focus group discussion took about 1 hour to complete.

As already reported above, HoDs were interviewed individually. The interviews took 50 minutes to complete. The focus of the interview questions for the HoDs was to find out whether there were any noticeable changes in the academic performance of staff members in their departments which could be attributed to their participation in the MAL-ED project.

Qualitative data obtained from the postgraduate students, junior faculty and HoD interviews were analysed using the thematic content analysis approach.^17^ The principal researcher transcribed the audio-recorded data. Codes were generated and the emerging themes were recorded. The data were then fed into the Atlas.ti software version 7.5.10. The results of analysis were linked to the themes that had been identified earlier. The researchers deliberated on the outputs and reached consensus on the final themes and categories to be adopted.

## Results of the study

The participants’ perceptions on the various aspects of their human capital development as a result of active involvement in the MAL-ED project are described below and are presented one theme at a time. Research skills and knowledge, experience and exposure, interpersonal skills and personal development were the themes distilled from the results of the interviews and focus group discussion. Also addressed below are the expectations of postgraduate students and junior faculty regarding their participation in the biomedical community research project.

### Research skills and knowledge

Of the 22 postgraduate students and junior faculty, 18 agreed that participating in the project resulted in them being empowered with research skills and knowledge. Cognitive testing, conducting anthropometric tests, and collection of bio-specimens such as stools and urine were specifically cited as the knowledge and skills they acquired. Some junior faculty were happy that they had been entrusted with leadership and supervision roles, which included overseeing data collection, checking completed forms, reviewing assessments and training field workers. The satisfaction is evident in the following views:

*It was a privilege for me to be recruited for the project. The project brought a lot of things to my research. I learnt how to do cognitive testing, to get data from children, to adapt to new equipment and to collect quality data*. (Participant #14)

*I am now overseeing data collection. I do quality checking of forms, reviewing assessments and training of fieldworkers. I collaborate with the Data Coordinating Centre. I was promoted to a lecturer position because of what I learnt from the MAL-ED project. I have been called to many interviews because of the skills I now possess*. (Participant #13)

### Experience and exposure

A total of 14 of the 22 postgraduate students and junior faculty alluded to the fact that they had gained experience and valuable exposure. Some of the participants learnt how to relate with different people such as the mothers, children and community members involved in the studies. It was highlighted that the participants managed to put the theory they learnt at university into practice. Some participants attested to having gained experience in using laboratory equipment in addition to being exposed to the general ‘world’ of research. Some of these views are presented below:

*The project has taught me to put theory into practice. At school, things were theoretical but the project gave me the practical side of things. I learnt to conduct anthropometric measurements. I gained a lot of experience in the field. I learnt about how people react to issues. I learnt how to relate to people*. (Participant #1)

*The project is full of laboratory work, blood processing, fieldwork and ordering of laboratory supplies. I got a lot of experience in DNA extraction from stools and saliva. I also attended field meetings. I have gained a lot of experience and exposure to blood processing from the project. MAL-ED has widened my scope on many issues*. (Participant #12)

### Interpersonal skills

Another benefit that accrued to participants in the MAL-ED project was the enhancement of interactive skills. It was explained that the acquisition of interpersonal skills resulted in continuous communication with community members and colleagues. In the process, the participants in the project worked well as a team, and respected and appreciated their diversity in terms of people, ideas and cultures – which is crucial for fieldworkers because it helps create conditions that enable collection of data of high quality. Some participants articulated their experiences as follows:

*I have learnt how to relate to people and I have made friends in the process. I have learnt to communicate with different community members*. (Participant #2)

*I have learnt how to communicate well with community members and to understand community issues. I have learnt to interact with everybody, poor or not. I have a good relationship with the participating mothers. I know how to maintain good relationships with children*. (Participant #5)

### Personal development

It was revealed that the MAL-ED project helped expand the capabilities of those involved in implementing it, developed and strengthened their character and also motivated others to pursue further studies or advance seemingly stalled careers. These experiences highlight the fact that even though the participants were recruited to implement the project, they were empowered and learnt to enhance the capacities of others as well. Some respondents commented:

*The project has improved my confidence. I have learnt to work with different people of different cultures. I now understand how people respond to pressure and commitment. I have learnt to understand my work colleagues*. (Participant #3)

*The project helped me in my personal development. I also felt a sense of belonging. The project gave me enough ground to explore my capabilities. I was able to empower someone to take over from me. From MAL-ED, I learnt to write manuscripts, attend conferences and present papers and to interact with more experienced researchers. MAL-ED was like a family to me*. (Participant #22)

### Other expectations of postgraduate students and junior faculty

Despite the varied human capital benefits that accrued to the postgraduate students and junior faculty through their involvement in the MAL-ED project, 9 out of the 22 postgraduate students and junior faculty indicated that they needed further training on data analysis and interpretation, exposure to a wider range of laboratory techniques as well as time and project management training. Active listening, how to handle children and research techniques were other areas that were identified for further training. Some participants wanted to be involved in other laboratory procedures and in data analysis that was conducted downstream. The following sentiments shed more light on these results:

*An opportunity in analysing quantitative data and interpreting it would have helped me*. (Participant #8)

*I wish I could be exposed to other new laboratory techniques and attend more training workshops*. (Participant #12)

## Human capital development as perceived by heads of departments

In general, the HoDs interviewed in the current study corroborated the views expressed by junior faculty. They were of the view that research skills such as data collection, data analysis, writing of quality manuscripts and mentoring were enhanced. In addition, they believed that the project assisted the participants in their personal development. Some of the members of staff seemed to collaborate with other scientists much better than they had before. They demonstrated more mature behaviour in the way they executed their work, with some gaining promotion mainly because of the quality of their research outputs as a result of their participation in the MAL-ED project. Below are some of the views of the HoDs:

*The multi-disciplinary approach of the project (microbiology, psychology and nutrition) exposes one to different people and a different way of looking at a problem. The project exposed people to practical ways of understanding issues. Staff members have shown maturity in the way they manage focus group discussions, transcribing, collecting data and writing articles*. (Participant #25)

*Research related skills have improved. There has been improvements in the skill of data collection, experience in research protocols like community entry and ethical issues. The skill of data collection has been enhanced. Staff members have been exposed to manuscript writing*. (Participant #23)

*The quality of publications are much improved in the sense that works were accepted in high profile journals which in a way attests to the quality of the work. Some members of staff have become professors based on the output from the project. This has enhanced their careers*. (Participant #25)

### Consolidated views from individual in-depth interviews, focus group and heads of departments

The most prominent responses drawn from individual in-depth interviews are presented in Table 1. It should be noted that the results obtained through individual interviews and focus group discussions were virtually the same. In general, the project contributed to considerable human capital development among postgraduate students and junior faculty.

## Discussion

Participation of postgraduate students and junior faculty in the MAL-ED biomedical community research project was found to have particularly enhanced their skills and knowledge. The projects that postgraduate students and junior academic staff mount, the lectures prepared for delivery and manuscripts written for publication in scientific media depend on and demand that quality research be undertaken. A considerable number of the postgraduate students and junior faculty who participated in the MAL-ED project secured jobs in the public sector, highlighting the immense value and capacity enhancing power of participating in the project. Other participants explained that, as a result of their involvement in the project, they were promoted to senior positions. This is in agreement with Ibok and Ibanga’s^18^ contention that improvements in skills and knowledge increases individuals’ employment prospects and related size of remuneration packages. The improved employability of the research participants is worth highlighting, especially considering the high levels of unemployment in the country. These results suggest that the quality of postgraduate programmes can be improved if students are integrated into ongoing research projects that senior academics run. Thus, it is prudent to take this into account in the course of designing innovative curricula for research-based postgraduate degrees.

The participants’ views in the current study resonate with those expressed by the grassroots community members in Moyo et al.’s^16^ study conducted in the community in which the MAL-ED project was implemented. Acquisition of knowledge and research skills enhanced personal development and enabled the grassroots community members in the forefront of project implementation to secure jobs. A study by Dongre et al.^19^ in India which focused on the benefits of exposing medical undergraduate students to community-based surveys yielded similar results to those of the current study. The benefits that accrued to the medical students included the ability to conduct interviews, better communicate with local villagers, collect and enter or store data using computers, and apply learning to research work as well as enhanced awareness of the public health process.

Postgraduate students and junior faculty also reported that the project enabled them to gain experience and exposure to new research techniques and practical realities. For instance, they pointed out that the project had enabled them to apply the theory they had learnt at university to real-life situations. This enhanced their understanding and appreciation of key concepts that might have otherwise remained abstract. Many participants were exposed to the realities of community-based research for the first time through this initiative. They had the opportunity to work with and interact with children and their mothers together with other grassroots community members for the first time. This helped to build and strengthen their social relationships and networking and enhanced their communication skills. In the process, the research participants acquired valuable experience and exposure, resulting in them gaining confidence in what they do. These views corroborate the observations of Tingen et al.^20^ in a study in Atlanta (Georgia, USA).

In the latter study, undergraduate nursing students gained professional experience and personal development through their participation in a research project conducted by their faculty. As was the case with the MAL-ED South Africa project, the nursing students in Tingen et al.’s^20^ study had limited exposure to research processes prior to their involvement in the study.

The students revealed that their involvement in the project improved their research skills, in particular data review, analysis and writing manuscripts for scholarly publishing. This reinforced the widely held notion that ‘experience is the best teacher’. Another study in Japan^21^ highlighted the benefits of exposing health science students to off-campus education. Students were able to interact with local residents. Afterwards, they expressed positive views about their community-based experience. Thus, exposing students to real-life environments enhances their understanding of issues and is likely to result in better graduate attributes.

Research participants cited enhancement of interpersonal skills as another attribute of human capital development gained through the current study. They mentioned improved ability to communicate well with grassroots community members and peers and researching together with mothers and their children as they collected time series data. As they participated in the project and remained in constant contact with the participants and grassroots community members, they became less judgmental of others and learnt how best to interact with a diverse range of people. Having an accommodating personality helps improve social relationships and cohesion – all of which are important prerequisites for individual and collective development. Raman^22^ concurs with this viewpoint and emphasises that effective interpersonal communication skills are a prerequisite of social interaction and building sustainable relationships.

Lastly, personal development was achieved through creating opportunities for the participants to apply the theory they had learnt in class to real-life situations, thereby boosting their confidence. When an individual’s confidence is boosted, they often feel motivated to perform better in the tasks assigned to them. Ultimately, they improve the performance of the organisation for which they work. The fact that some participants became so motivated that they eventually pursued further studies was crucial because it helped build their professional careers. Their desire to improve their educational qualifications resonates with the ideals of the late Nelson Mandela who alluded to the fact that education is the most powerful weapon which one can use to transform oneself and the world around them. Others reported that they were better organised, more disciplined and were able to work more independently as a result of their involvement in the MAL-ED project. All these are positive attributes that demonstrate maturity, which their HoDs confirmed to be the case. Thus, it can be concluded that participation in the MAL-ED project enabled both postgraduate students and junior faculty to unlock and sharpen their capabilities.

## Limitations of the study

Although only 25 and 10 people participated in the interviews and focus group discussions, respectively, their insights into the benefits accruing to them as a result of their involvement in the MAL-ED project were quite revealing. Ideally, having more than one focus group could have enabled deeper, independent interrogation of issues. However, the fact that the results of the individual interviews and focus group discussion were virtually the same, highlighted the fact that what is reported here was a true reflection of participants’ experiences.

## Conclusions

Skills and knowledge in research, experience and exposure, interpersonal skills and personal development were the major benefits that accrued to postgraduate students and junior faculty as a result of their participation in the biomedical community research project. Some participants highlighted the need for training workshops that focused on data analysis and interpretation, exposure to other laboratory techniques as well as time and project management training. These findings are useful as inputs into the planning and implementation of future similar projects.

## Figures and Tables

**Table 1:** Consolidated views from individual in-depth interviews, the focus group discussion and individual heads of department interviews

Benefits	In-depth interviews	Postgraduate students (PS)/ junior faculty (JF)	Focus group	Heads of department
1. Experience in working with different people	The project helped me to deal with people of different cultures. (8)	PS	•	••
2. Other benefits	I benefitted financially. I used the money to further my studies. (8)	PS	•	••
3. Research knowledge and acquisition of research skills	The study brought a lot of insights about research, initial preparation for a study and the reason for carrying out a study. (22)	PS and JF	•	••
4. Personal development	The study motivated me to study further. (21)	PS and JF	•	••
5. Writing scientific research manuscripts	The project has helped me in writing manuscripts. At times we wrote as a team.(7)	JF	•	••

Perspectives from individual interviews regarding the benefit statements are shown in the first column. Responses to those statements by the respondents are indicated in numerals in the second column. The third column shows the group of participants who responded to each of the key statements. A bullet (•) in the fourth column is used to indicate that each response was supported by the focus group. The number of bullets in the heads of departments column represents the number of heads of departments who selected each of the statements.

## Significance:

- The importance of engaging study participants to get their views on the ‘hidden’ value of biomedical research projects has been identified.
- The need for community-based researchers to equip members of their teams with relevant research skills and knowledge has been emphasised.
- The results of this study can be useful in the planning and implementation of similar projects in the future.
